# Association between 24-h urinary sodium and potassium excretion and blood pressure among Chinese adults aged 18–69 years

**DOI:** 10.1038/s41598-021-83049-8

**Published:** 2021-02-10

**Authors:** Xiaofu Du, Le Fang, Jianwei Xu, Xiangyu Chen, Yamin Bai, Jieming Zhong

**Affiliations:** 1 Zhejiang Provincial Center for Disease Control and Prevention, 3399 Binsheng Road, Hangzhou, 310051 China; 2 National Center for Chronic and Noncommunicable Disease Control and Prevention, Chinese Center for Disease Control and Prevention, 27 Nanwei Road, Beijing, 100050 China

**Keywords:** Risk factors, Cardiovascular diseases

## Abstract

The direction and magnitude of the association between sodium and potassium excretion and blood pressure (BP) may differ depending on the characteristics of the study participant or the intake assessment method. Our objective was to assess the relationship between BP, hypertension and 24-h urinary sodium and potassium excretion among Chinese adults. A total of 1424 provincially representative Chinese residents aged 18 to 69 years participated in a cross-sectional survey in 2017 that included demographic data, physical measurements and 24-h urine collection. In this study, the average 24-h urinary sodium and potassium excretion and sodium-to-potassium ratio were 3811.4 mg/day, 1449.3 mg/day, and 4.9, respectively. After multivariable adjustment, each 1000 mg difference in 24-h urinary sodium excretion was significantly associated with systolic BP (0.64 mm Hg; 95% confidence interval [CI] 0.05–1.24) and diastolic BP (0.45 mm Hg; 95% CI 0.08–0.81), and each 1000 mg difference in 24-h urinary potassium excretion was inversely associated with systolic BP (− 3.07 mm Hg; 95% CI − 4.57 to − 1.57) and diastolic BP (− 0.94 mm Hg; 95% CI − 1.87 to − 0.02). The sodium-to-potassium ratio was significantly associated with systolic BP (0.78 mm Hg; 95% CI 0.42–1.13) and diastolic BP (0.31 mm Hg; 95% CI 0.10–0.53) per 1-unit increase. These associations were mainly driven by the hypertensive group. Those with a sodium intake above about 4900 mg/24 h or with a potassium intake below about 1000 mg/24 h had a higher risk of hypertension. At higher but not lower levels of 24-h urinary sodium excretion, potassium can better blunt the sodium-BP relationship. The adjusted odds ratios (ORs) of hypertension in the highest quartile compared with the lowest quartile of excretion were 0.54 (95% CI 0.35–0.84) for potassium and 1.71 (95% CI 1.16–2.51) for the sodium-to-potassium ratio, while the corresponding OR for sodium was not significant (OR, 1.28; 95% CI 0.83–1.98). Our results showed that the sodium intake was significantly associated with BP among hypertensive patients and the inverse association between potassium intake and BP was stronger and involved a larger fraction of the population, especially those with a potassium intake below 1000 mg/24 h should probably increase their potassium intake.

## Introduction

Elevated BP has become a major risk factor for the global burden of disease, and the resulting stroke and heart disease are the leading causes of death and DALYs at the national level in China^1,2^. The latest China Hypertension Survey showed that the prevalence of hypertension has increased from 18.0 to 23.2%^3^, and approximately 244.5 million individuals in the Chinese adult population have hypertension, whereas approximately 435.3 million individuals (41.3%) have prehypertension according to the Chinese guidelines^4^. Various studies have suggested that high sodium and low potassium are among the key risk factors for hypertension^5,6^. The daily salt intake in China and worldwide is 10.5 g/day^7^ and 9–12 g/day^8,9^, respectively, which is far beyond the recommended amount by the Chinese Dietary Guidelines (less than 6 g/day)^10^ and the World Health Organization (less than 5 g/day)^11^. Furthermore, the potassium intake of the Chinese population is 1.42 g/day, less than half of the World Health Organization (WHO) recommended amount of > 3.5 g/day^12^. Therefore, reducing sodium intake and increasing potassium intake in the general population has been considered a cost-effective action that should be maintained and expanded to save lives, prevent diseases and reduce healthcare costs^13,14^.

The high sodium and low potassium intake in China require change. The relationship between sodium and potassium intake and BP is the basis for formulating strategies for reducing sodium and supplementing potassium to lower BP. These accurate and in-depth relationship studies benefit from multiple complete 24-h urine collections rather than food frequency methods or questionnaire survey methods because they are not restricted by high variability of dietary intake patterns or recall bias. In addition, the difference between the various results of relationship research lies in the magnitude of the association with BP. Other studies have shown that the magnitude of BP lowering through sodium reduction was greater for those with higher baseline BP, which also indicates that the association was larger among hypertensive populations than among normotensive persons^15,16^. Overall, evidence has shown that the association between sodium, potassium, and their ratio with BP was affected by ethnicity, BMI, dietary sodium and potassium consumption level, hypertensive status, and other essential anthropometric characteristics^17^. At present, population-based studies of the associations between sodium or potassium intake and BP or hypertension among Chinese adults are limited. In this study, we used cross-sectional survey data from the Salt Reduction and Hypertension Prevention Project (SRHPP) in Zhejiang Province, China to estimate the associations of sodium or potassium and their ratio with BP or hypertension among Chinese adults, adjusting for within-person variability in 24-h urinary electrolyte excretion and potential confounding variables.

## Methods

### Design

Cross-sectional survey data were analyzed from the SRHPP baseline survey in Zhejiang Province, China in 2017, which aimed to study hypertension and salt intake and to provide salt reduction strategies. Participants aged 18–69 years living in the selected areas for 6 to 12 months before the investigation were recruited by a stratified multistage random sampling method. Briefly, 5 project points containing 2 rural areas and 3 urban areas distributed in the east, northeast, central, midwest, and south of Zhejiang Province were included in the study to achieve balance and representativeness. A roster was set up in each village or neighborhood committee selected, and qualified residents were selected and mobilized face to face to participate in the project. Eventually, a provincially representative sample of 7512 residents gave informed consent and participated in the project with a complete database, of which 1424 responders collected complete 24-h urine to assess the overall sodium intake. Methods for sample size calculation and sample selection are described in detail elsewhere^18^. The permission protocol to undertake the SRHPP and written participant consent were obtained from the China Centers for Disease Control and Prevention (CDC) and all participants, respectively. The project was approved by the Zhejiang Provincial CDC Ethics Review Committee. All experiments, including questionnaire surveys, physical examinations and biological specimen testing, were performed in accordance with relevant guidelines and regulations.

All the respondents were invited to participate in a close-ended face-to-face survey by trained technicians. Questionnaire investigation was used to collect information on participants' demographic characteristics, history of hypertension, diabetes and CVD, as well as the lifestyle habits of smoking, alcohol use, and physical activity, which were applied as adjustment variables for association studies. Educational attainment was divided into 3 categories according to the years of education (< 9 years, 9–12 years, or > 12 years). A self-reported history of CVD included coronary heart disease and stroke, and diabetes status and chronic kidney disease (CKD) were determined by self-reported diagnosis from a healthcare provider or the use of related medication. Former smoker was defined as a total of more than 100 cigarettes smoked and current smoker was defined as an average of more than 1 cigarette per day for 6 consecutive months; participants were otherwise defined as nonsmoker. Self-reported physical activity referred to ≥ 150 min per week of moderate-intensity or a combination of moderate- and high-intensity exercise or ≥ 75 min per week of high-intensity exercise. Alcohol consumption referred to those who drink ≥ 1 time a week in the past year. Alcoholic beverages included beer, liquor, red wine, and rice wine.

Every individual sampled had a physical examination comprising assessment of BP, weight, and height.

### Main outcomes and measures

The 24-h urine sample was used to calculate the sodium and potassium excretion. In this study, stratified according to age group (5 levels) and sex (2 levels), 1512 individuals were randomly selected from all 7512 participants for an additional 24-h urine collection. During the investigation, a leaflet with explanations along with the necessary equipment (3 L standard urine collection container) was provided to the participants and 24-h urine collection procedures were explained^19^, i.e., urinate upon waking on the first day, discard that sample and then collect all urine including that voided upon waking the next day. At the urine collection site, the 24-h urine volume was measured, and the start and end times of urine collection were recorded. An aliquot (5 ml) of the sample was taken and shipped using a cold chain to a central lab (KingMed Diagnostics Laboratory Inc., Hangzhou, China) for sodium, potassium and creatinine assays, with the remainder discarded. An ion selective electrode method was used for sodium and potassium analysis (C16000, Abbott Corp., America), and the picric acid method was used for creatinine analysis (C501, Roche Cob as Corp., Switzerland). The excretion content of each indicator was obtained from the test concentration multiplied by the 24-h urine volume. Urine specimens were considered incomplete if the collection time was less than 22 h, total urine volume was less than 500 ml, or 24-h urinary creatinine excretion was ± 2 standard deviations outside of the sex-specific mean^20,21^. Of the participants selected for 24-h urine collection, 1424 (94.2% of 1512) returned a complete specimen.

Systolic blood pressure (SBP) and diastolic blood pressure (DBP) measurements were performed according to internationally accepted measurement methods and quality control specifications in triplicate with the participant seated after 5 min of rest. Measurements were recorded at least one minute apart using a validated automatic electronic sphygmomanometer (HEM-7071, Omron Corp., Japan) with an appropriately sized cuff, and the average value of the three measurement results was taken. The participants were divided into 3 categories based on BP and antihypertensive medication usage. Hypertension was defined as an average SBP ≥ 140 mm Hg and/or an average DBP ≥ 90 mm Hg and/or self-reported use of antihypertensive medication within 2 weeks. Prehypertension was defined as an average SBP of 120–139 mm Hg or an average DBP of 80–89 mm Hg, and normotension was defined as an average SBP < 120 mm Hg and an average DBP < 80 mm Hg without antihypertensive medication^22^. The average SBP and DBP (MBP, i.e., SBP + DBP divided by 2), considered the most informative measure for stroke mortality and heart disease mortality, was also examined^23^.

### Statistical analysis

The baseline characteristics of the participants were summarized as proportions and means (SD, standard deviation). ANOVA for continuous variables and the χ^2^ test for categorical variables were used to compare the demographic and health characteristics across hypertensive status categories. ANOVA adjusted for age, sex, ethnicity, and body mass index (BMI) was used to compare least-squares means of 24-h urinary sodium and potassium excretion and the sodium-to-potassium ratio. The preliminary adjusted model mainly adjusted for age, sex and ethnicity. The fully adjusted model additionally included BMI, education level, history of CVD, diabetes, CKD, smoking status, alcohol use and physical activity. Potassium excretion is controlled in the models of sodium excretion, and vice versa, and the models for the sodium-to-potassium ratio did not adjust for sodium and potassium excretion. We used multivariable linear regression to assess the associations of BP with 24-h urinary sodium and potassium excretion (each 1000 mg) and the sodium-to-potassium ratio (each 1-unit molar ratio). Due to potential misclassification at the quartile cut points leading to variability in sodium and potassium excretion, we compared BP across population quartiles of sodium and potassium excretion and the sodium-to-potassium ratio after we calculated the 12.5th, 37.5th, 62.5th, and 87.5th percentiles distribution of estimated usual excretion and estimated the adjusted SBP and DBP of these percentiles using the parameters from multivariable linear regression models based on the approximately linear relationship between sodium, potassium, and the sodium-to-potassium ratio and BP. To show the interaction of sodium and potassium on BP, we determined the relationship between sodium and potassium and adjusted SBP and DBP in different quartiles. For hypertensive status, multivariable logistic regression was used to assess associations with odds of hypertension from quartiles of sodium or potassium (at the mid-values of each excretion quartile, Q4, Q3 and Q2 versus the lowest quartile Q1), comparing the adjusted odds for normotension and prehypertension versus hypertension. We conducted several sensitivity analyses as follows: (1) restricted to individuals not taking antihypertensive medications (n = 1220); (2) restricted to individuals without CVD (n = 1391); (3) excluding alcohol consumption from covariates (n = 1424); and (4) excluding BMI from covariates (n = 1424). Statistical analyses were performed with SPSS for Windows (Version 26, SPSS Inc, Chicago, IL). *P* values < 0.05 were deemed significant.

## Results

In our study, the average participant age was 46.7 (14.1) years, 51.1% were female, 98.7% were of Han ethnicity, and over 2/3 were classified as hypertensive (36.3%) or prehypertensive (33.5%). The prevalence of hypertension was higher among males than females and higher in rural areas than in urban areas. Participants with hypertension generally had a higher age, lower education attainment, higher BMI, and higher smoking and drinking rates; a higher proportion of participants with hypertension also had diabetes mellitus, CVD, and CKD than those with prehypertension or normotension (all *P* < 0.05, Table 1). In addition, the 24-h urinary sodium and potassium excretion of 1424 participants with urinary integrity were 3811.4 (1644.4) mg/day and 1449.3 (653.6) mg/day, respectively. Sodium did not differ by hypertensive status or household registration type after adjustment for age, sex, ethnicity, and BMI. However, there was a significant difference in potassium; that is, the normotensive group had better potassium levels than the other two groups (Table 2).

**Table 1:** Participant characteristics by hypertensive status among Chinese adults aged 18 to 69 years.

Characteristic	All objects (n = 1424)	Normotensive (n = 430)	Prehypertensive (n = 477)	Hypertensive (n = 517)	*P* value
Age, year	46.7 ± 14.1	38.9 ± 12.7	45.0 ± 13.9	54.7 ± 10.7	< 0.001*
**Gender, n (%)**					< 0.001*
Male	697 (48.9)	141 (32.8)	269 (56.4)	287 (55.5)	
Female	727 (51.1)	289 (67.2)	208 (43.6)	230 (44.5)	
**Ethnicity, n (%)**					0.749
Han	1405 (98.7)	423 (98.4)	472 (99.0)	510 (98.6)	
Others	19 (1.3)	7 (1.6)	5 (1.0)	7 (1.4)	
**Household registration type, n (%)**					0.296
Urban	621 (43.6)	178 (41.4)	204 (42.8)	239 (46.2)	
Rural	803 (56.4)	252 (58.6)	273 (57.2)	278 (53.8)	
**Education, n (%)**					< 0.001*
< 9 years	480 (33.7)	98 (22.8)	139 (29.1)	243 (47.0)	
9–12 years	656 (46.1)	188 (43.7)	237 (49.7)	231 (44.7)	
> 12 years	288 (20.2)	144 (33.5)	101 (21.2)	43 (8.3)	
BMI, kg/m^2^	24.0 ± 3.3	22.5 ± 2.9	23.9 ± 3.1	25.5 ± 3.2	< 0.001*
Stroke, n (%)	17 (1.2)	0 (0.0)	4 (0.8)	13 (2.5)	0.001*
Coronary heart disease, n (%)	17 (1.2)	2 (0.5)	4 (0.8)	11 (2.1)	0.044*
Diabetes mellitus, n (%)	132 (9.3)	15 (3.5)	21 (4.4)	96 (18.6)	< 0.001*
Self-report kidney disease, n (%)	7 (0.5)	0 (0.0)	0 (0.0)	7 (1.4)	0.002*
**Smoking status, n (%)**					< 0.001*
Never smoked	1035 (72.7)	358 (83.3)	330 (69.2)	347 (67.1)	
Former smoker	66 (4.6)	14 (3.3)	18 (3.8)	34 (6.6)	
Current smoker	323 (22.7)	58 (13.5)	129 (27.0)	136 (26.3)	
Alcohol use status, n (%)	465 (32.7)	113 (26.3)	180 (37.7)	172 (33.3)	0.001*
Physical activity, n (%)	578 (40.6)	180 (41.9)	187 (39.2)	211 (40.8)	0.713
24-h creatinine, mg/24 h	1077.6 ± 440.7	1054.2 ± 435.4	1124.1 ± 443.5	1054.1 ± 439.8	0.018*
24-h urine volume, ml/24 h	1443.0 ± 441.5	1388.0 ± 445.9	1440.0 ± 423.1	1491.4 ± 449.7	0.002*

^a^Sample sizes (n), means and prevalence are unweighted.

*P < 0.05.

**Table 2:** Estimated mean 24-h urinary sodium and potassium excretion and their ratio among participants.

	All objects (n = 1424)	Normotensive (n = 430)		Prehypertensive (n = 477)		Hypertensive (n = 517)		*P* value
	Mean	Mean	SE	Mean	SE	Mean	SE	
Sodium excretion, mg/24 h	3811.4	3891.8	86.1	3772.7	74.0	3776.0	78.7	0.537
Potassium excretion, mg/24 h	1449.3	1511.0	34.9	1478.3	29.9	1369.2	31.8	0.012*
Sodium-to-potassium ratio	4.9	4.8	0.1	4.8	0.1	5.2	0.1	0.069

Estimated means of usual excretion were adjusted for age, sex, ethnicity, and body mass index. F tests in 24-h urinary potassium excretion and sodium-to-potassium ratio by hypertensive status were significant. The ratio of sodium-to-potassium was expressed as a molar ratio. SE, standard error.

*P < 0.05.

**Figure 1:**
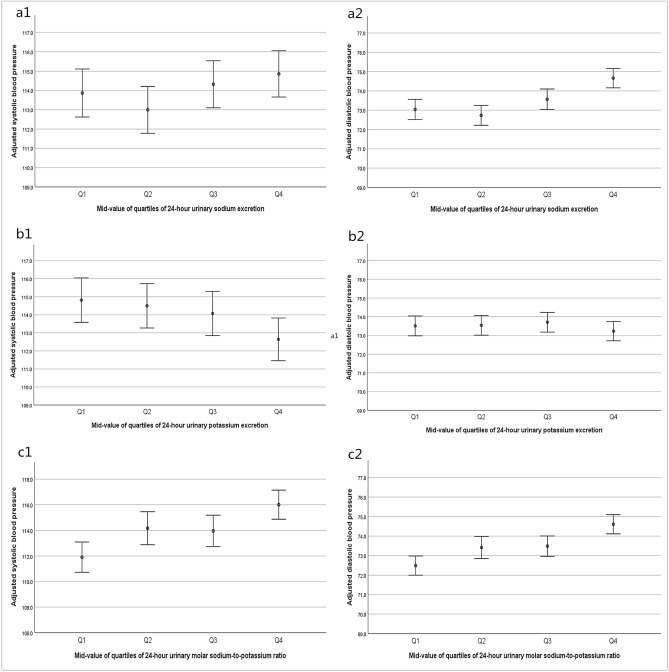
Adjusted SBP and DBP at the mid-quartile of 24-h urinary sodium and potassium excretion and their ratio. This figure presents adjusted SBP at the 12.5th, 37.5th, 62.5th, and 87.5th percentiles of sodium excretion (**a1**), potassium excretion (**b1**), and the sodium-to-potassium ratio (**c1**). Simultaneously, the results related to DBP are shown in (**a2**), (**b2**), and (**c2**).

After full adjustment (Fig. 1), the adjusted average SBP and DBP were higher with increasing quartiles of sodium excretion and ratio of sodium-to-potassium (*P* < 0.05), and at Q2 of sodium excretion, the adjusted SBP and DBP were relatively lower. The adjusted average SBP and DBP were lower across the higher quartiles of potassium excretion (*P* < 0.05).

In fully adjusted linear regression models (Table 3), sodium excretion was directly associated with SBP (0.64 mm Hg; 95% CI 0.05–1.24), DBP (0.45 mm Hg; 95% CI 0.08–0.81), and MBP (0.54 mm Hg; 95% CI 0.10–0.99) for each 1000 mg increase in sodium intake. Potassium excretion was inversely associated with SBP (− 3.07 mm Hg; 95% CI − 4.57 to − 1.57), DBP (− 0.94 mm Hg; 95% CI − 1.87 to − 0.02) and MBP (− 2.01 mm Hg; 95% CI − 3.13 to − 0.89) for each 1000 mg increase in potassium intake. The molar ratio of sodium-to-potassium was directly associated with SBP (0.78 mm Hg; 95% CI 0.42–1.13), DBP (0.31 mm Hg; 95% CI 0.10–0.53) and MBP (0.55 mm Hg; 95% CI 0.28–0.81) per 1 unit increase. In the study of the association between sodium, potassium and BP in the hypertension subgroup, the overall significant relationship was attributed to hypertensive patients because the relationship in normotensive or prehypertensive participants did not reach significance.

**Table 3:** Association between 24-h urinary sodium and potassium excretion and their ratio with blood pressure.

	SBP	DBP	MBP
	β-coefficient (95% CI)^a^	β-coefficient (95% CI)^a^	β-coefficient (95% CI)^a^
**All subjects, n = 1424**			
**Sodium excretion**			
Adjusted for age, sex, ethnicity^b^	1.14* (0.52 to 1.76)	0.80* (0.41 to 1.18)	0.97* (0.50 to 1.44)
Fully adjusted model^c^	0.64* (0.05 to 1.24)	0.45* (0.08 to 0.81)	0.54* (0.10 to 0.99)
**Potassium excretion**			
Adjusted for age, sex, ethnicity^b^	− 3.23* (− 4.77 to − 1.68)	− 1.02* (− 1.97 to − 0.06)	− 2.12* (− 3.29 to − 0.95)
Fully adjusted model^c^	− 3.07* (− 4.57 to − 1.57)	− 0.94* (− 1.87 to − 0.02)	− 2.01* (− 3.13 to − 0.89)
**Sodium-to-potassium ratio**			
Adjusted for age, sex, ethnicity^b^	0.88* (0.51 to 1.24)	0.38* (0.15 to 0.61)	0.63* (0.35 to 0.91)
Fully adjusted model^c^	0.78* (0.42 to 1.13)	0.31* (0.10 to 0.53)	0.55* (0.28 to 0.81)
**Normotensive, n = 430**			
**Sodium excretion**			
Adjusted for age, sex, ethnicity^b^	− 0.05 (− 0.49 to 0.39)	0.15 (− 0.23 to 0.53)	0.05 (− 0.31 to 0.40)
Fully adjusted model^c^	− 0.22 (− 0.67 to 0.22)	0.02 (− 0.37 to 0.41)	− 0.10 (− 0.46 to 0.26)
**Potassium excretion**			
Adjusted for age, sex, ethnicity^b^	0.35 (− 0.76 to 1.47)	0.66 (− 0.31 to 1.62)	0.51 (− 0.40 to 1.41)
Fully adjusted model^c^	0.30 (− 0.84 to 1.44)	0.68 (− 0.31 to 1.67)	0.49 (− 0.43 to 1.41)
**Sodium-to-potassium ratio**			
Adjusted for age, sex, ethnicity^b^	− 0.01 (− 0.30 to 0.28)	− 0.07 (− 0.32 to 0.18)	− 0.04 (− 0.27 to 0.19)
Fully adjusted model^c^	− 0.05 (− 0.34 to 0.24)	− 0.12 (− 0.37 to 0.14)	− 0.08 (− 0.32 to 0.15)
**Prehypertensive, n = 477**			
**Sodium excretion**			
Adjusted for age, sex, ethnicity^b^	− 0.36 (− 0.76 to 0.05)	0.25 (− 0.13 to 0.64)	− 0.05 (− 0.35 to 0.24)
Fully adjusted model^c^	− 0.46* (− 0.88 to − 0.04)	0.12 (− 0.27 to 0.52)	− 0.17 (− 0.47 to 0.13)
**Potassium excretion**			
Adjusted for age, sex, ethnicity^b^	− 0.99* (− 1.94 to − 0.04)	0.04 (− 0.87 to 0.94)	− 0.48 (− 1.17 to 0.22)
Fully adjusted model^c^	− 0.95 (− 1.93 to 0.03)	− 0.19 (− 1.10 to 0.72)	− 0.57 (− 1.27 to 0.13)
**Sodium-to-potassium ratio**			
Adjusted for age, sex, ethnicity^b^	0.02 (− 0.20 to 0.24)	0.04 (− 0.17 to 0.24)	0.03 (− 0.13 to 0.18)
Fully adjusted model^c^	− 0.01 (− 0.23 to 0.22)	0.03 (− 0.17 to 0.24)	0.01 (− 0.15 to 0.17)
**Hypertensive, n = 517**			
**Sodium excretion**			
Adjusted for age, sex, ethnicity^b^	2.01* (0.99 to 3.04)	0.94* (0.34 to 1.55)	1.48* (0.75 to 2.20)
Fully adjusted model^c^	1.40* (0.37 to 2.44)	0.59 (− 0.03 to 1.21)	1.00* (0.26 to 1.73)
**Potassium excretion**			
Adjusted for age, sex, ethnicity^b^	− 4.40* (− 7.04 to − 1.76)	− 1.20 (− 2.76 to 0.36)	− 2.80* (− 4.67 to − 0.93)
Fully adjusted model^c^	− 3.05* (− 5.74 to − 0.36)	− 0.55 (− 2.15 to 1.06)	− 1.80 (− 3.70 to 0.10)
**Sodium-to-potassium ratio**			
Adjusted for age, sex, ethnicity^b^	1.56* (0.95 to 2.18)	0.55* (0.18 to 0.91)	1.05* (0.62 to 1.49)
Fully adjusted model^c^	1.22* (0.58 to 1.85)	0.36 (− 0.02 to 0.74)	0.79* (0.34 to 1.24)

^a^β-coefficients for sodium and potassium indicate the change in mm Hg of blood pressure associated with per 1000 mg difference in excretion; β-coefficients for the ratio of sodium-to-potassium indicate the change in mm Hg of blood pressure associated with each 1-unit increase in molar ratio.

^b^Preliminary adjusted model including age, sex, and ethnicity.

^c^Fully adjusted models included age, sex, and ethnicity plus body mass index, education level, history of cardiovascular disease, diabetes mellitus status, self-reported chronic kidney disease, antihypertensive medication use, smoking status, alcohol use status, and physical activity. In addition, sodium intake was adjusted in the regression models for potassium, and vice versa, and the models for the sodium-to-potassium ratio did not adjust for sodium and potassium excretion.

*P < 0.05 for β-coefficient in the regression model.

To have more effective and stable public health and clinical guidance, hypertension status was a binary variable (hypertension and non-hypertension), and binary logistic regression analysis was performed in this section. In the preliminary adjusted multivariable logistic model (Table 4), the association between sodium intake and hypertension was only significant in the Q4 of sodium intake, and the cutoff value was about 4900 mg/24 h (that is, the middle value of Q3 and Q4 of sodium excretion). In the fully adjusted multivariable logistic model, there seemed to be a clinically relevant inverse association between potassium intake and hypertension, and the cutoff value was about 1000 mg/24 h (that is, the middle value between Q1 and Q2 of potassium excretion). For example, individuals in the highest quartile, Q4, had a 0.46 times lower odds of having hypertension (OR 0.54; 95% CI 0.35–0.84) in comparison with the lowest quartile of potassium excretion, Q1, while correspondingly having 1.71 times higher odds of obtaining hypertension (OR, 1.71; 95% CI 1.16–2.51) from the sodium-to-potassium ratio Q4 in contrast to Q1. However, the association between sodium excretion and hypertensive status did not reach significance and the sodium-to-potassium ratio did not contribute with additional outcomes, just simply reflecting the outcome of the potassium intake.

**Table 4:** Associations of 24-h urinary sodium and potassium excretion and their ratio with hypertension.

	Q1 (12.5th percentile)	Q2 (37.5th percentile)	Q3 (62.5th percentile)	Q4 (87.5th percentile)
	OR, 95% CI	OR, 95% CI	OR, 95% CI	OR, 95% CI
Sodium excretion, mg/24 h^a^	1996	3096	4106	5715
Preliminary adjusted model^b^	1.00	0.99 (0.69–1.41)	1.04 (0.72–1.51)	1.53* (1.02–2.28)
Fully adjusted model^c^	1.00	0.92 (0.63–1.36)	0.92 (0.62–1.36)	1.28 (0.83–1.98)
Potassium excretion, mg/24 h^a^	781	1179	1542	2164
Preliminary adjusted model^b^	1.00	0.65* (0.45–0.93)	0.72 (0.50–1.05)	0.63* (0.43–0.94)
Fully adjusted model^c^	1.00	0.58* (0.39–0.86)	0.61* (0.41–0.92)	0.54* (0.35–0.84)
Sodium-to-potassium ratio^a^	2.71	3.94	5.19	7.22
Preliminary adjusted model^b^	1.00	1.54* (1.09–2.17)	1.48* (1.04–2.09)	1.75* (1.23–2.49)
Fully adjusted model^c^	1.00	1.37 (0.95–1.99)	1.37 (0.94–2.00)	1.71* (1.16–2.51)

Here, hypertension status was a binary variable (hypertension and non-hypertension), and binary logistic regression analysis was performed.

*Indicates *P* < 0.05.

^a^These rows contain the estimated mid-value of quartiles in the population.

^b^Preliminary adjusted model included age, sex, and ethnicity.

^c^Fully adjusted models included age, sex, ethnicity, education, body mass index, history of cardiovascular disease, mellitus status, chronic kidney disease, smoking status, alcohol use status and physical activity. In addition, models examining sodium excretion were simultaneously adjusted for potassium excretion, and vice versa.

**Figure 2:**
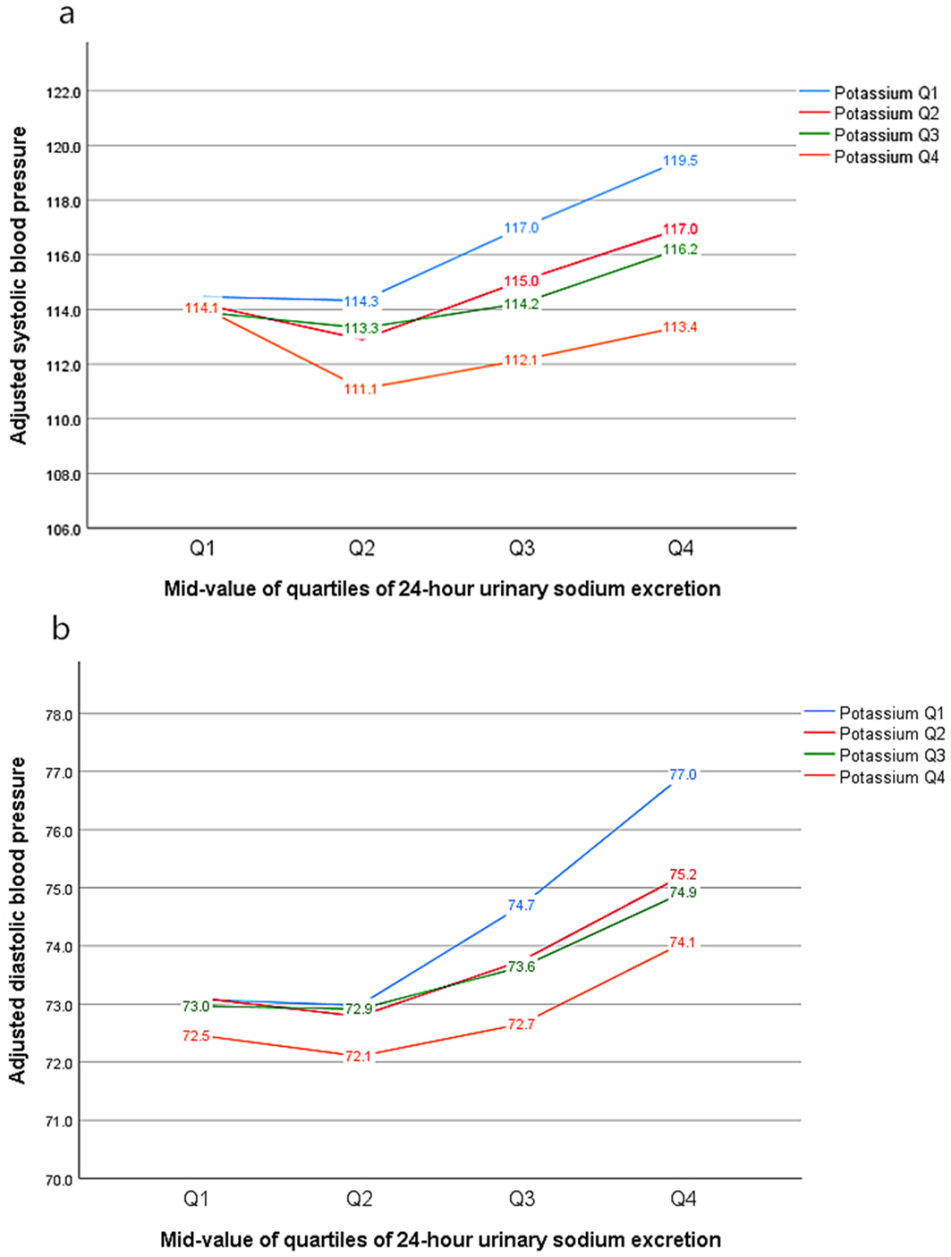
Adjusted SBP and DBP in the quartiles of 24-h urinary sodium and potassium excretion. This figure presents adjusted systolic blood pressure (**a**) and adjusted diastolic blood pressure (**b**) at the 12.5th, 37.5th, 62.5th, and 87.5th percentiles of 24-h urinary sodium and by the quartiles of potassium excretion.

In our analyses, the adjusted average BP remained higher for individuals in the Q4 quartile than for those in the Q1 quartile of 24-h urinary sodium excretion. Across the quartiles of 24-h urinary sodium, there was obvious evidence that higher potassium intake may blunt the sodium-BP relationship. Therefore, for potassium Q4 vs. potassium Q1 among sodium Q4, SBP was lower by 6.1 mm Hg; the other three comparisons showed smaller differences: for potassium Q4 vs. Q1 for sodium Q3, SBP was lower by 4.9 mm Hg; for potassium Q4 vs. Q1 for sodium Q2, SBP was lower by 3.2 mm Hg; and for potassium Q4 vs. Q1 for sodium Q1, SBP was lower by only approximately 0.5 mm Hg (Fig. 2). Similar results appear in the DBP relationship study. The data suggested that the higher the 24-h urinary sodium, the greater the capacity of potassium intake to dampen the sodium-BP relationship.

Interactions between sodium, potassium, or the sodium-to-potassium ratio and model covariates were not statistically significant after correction for multiple comparisons. The results remained robust in the sensitivity analyses (see Supplemental Table 1).

## Discussion

This provincially representative cross-sectional study that has reference significance at the national level demonstrated a direct association between urinary electrolyte excretion and multiple measures of BP among Chinese adults using the first collection of 24-h urine in Zhejiang Province.

The study benefits from the collection and usage of 24-h urine to assess salt consumption. Salt intake of the Chinese population mainly lies in home cooking (approximately 80% of total intake)^24^, and the many sources of salt include condiments, including salt, soy source and monosodium glutamate, bean paste, pickles, and some other "invisible" salts that consumers do not notice (for example, plum, instant noodles, and biscuits). Therefore, the self-reported dietary salt survey is very unstandardized and uncertain. Twenty-four-hour urinary excretion is considered the gold standard for estimated sodium intake, and in comparison with self-reported dietary salt intake data, urinary sodium excretion does not depend on the accuracy of self-reporting or food composition tables, and approximately 95% of ingested sodium is excreted through urine^21^. Therefore, the results of our association study are more reliable than those derived from dietary sodium^25^.

In this research, sodium and the sodium-to-potassium ratio were both directly associated with BP, whereas potassium excretion was inversely associated with BP. These results are consistent with prior findings of urinary electrolyte excretion and BP in other studies comprising animal experiments, epidemiological studies, and clinical trials^5,21,26,27^. While the directions of the association are generally the same, the magnitude of the reported findings may differ. Our results showed that urinary potassium has a greater effect on BP than urinary sodium.

In our study, we found a positive but nonuniform association between sodium excretion and BP in comparison with the INTERSALT study and ASC study (Action on Salt China in 2018); our results showed that the slope of the overall association between 24-h urinary sodium excretion and BP (0.64 mmHg in SBP and 0.45 mmHg in DBP increased by each 1000 mg difference) was not exactly consistent with those of the INTERSALT and ASC studies (0.94 or 1.32 mmHg in SBP and 0.03 or 0.34 mmHg in DBP increased by each 1000 mg of sodium, respectively)^28,29^. In a recent meta-regression analysis of 133 randomized controlled trials, per 1000 mg of sodium reduction was associated with a 3.34 mmHg drop in SBP among persons with higher BP (above 131/78 mmHg SBP/DBP) or with a 0.63 mmHg drop in SBP among persons with lower BP (under 131/78 mmHg SBP/DBP)^16^. Although studies on gene polymorphisms have shown that Asians might be more salt sensitive^30,31^, the strength of an association among Asian populations reported in studies is relatively lower. For example, a Japanese study showed that an increase per 1000 mg of sodium was associated with a 1.6 mmHg higher SBP and a 1.0 mmHg higher DBP^32^. Nevertheless, for estimated potassium excretion, the magnitude of the overall inverse association with BP in our study was greater. The significant inverse relationship between potassium excretion and BP (3.1 mmHg in SBP and 0.9 mmHg in DBP decreased by each 1000 mg) is substantially steeper than those of the INTERSALT study (0.65 mmHg in SBP and 0.42 mmHg in DBP decreased by each 1000 mg of potassium). The reason for the steep association between potassium and BP in our study may be due to the relatively lower potassium intake, making the current population sensitive to increased potassium intake. The ASC study and SMASH program (Shandong–Ministry of Health Action on Salt and Hypertension in China) also found a stronger inverse relationship between potassium excretion and BP. According to the WHO, the ratio of sodium-to-potassium should be less than 1.0^33^. In the DASH diet, further decreases in average sodium intake (to 2300 and 1500 mg/day) and the ratio of sodium-to-potassium (to 0.5–0.3) provide additional benefits^34^. Similarly, our study found that potassium was significantly associated with the risk of hypertension, consistent with NHANES 2014 (National Health and Nutrition Examination Survey in the United States)^21^, which found that the ORs for comparisons of the prevalence of hypertension among adults in the highest quartile versus the lowest quartile were 4.2 (95% CI 1.4–13.2) for sodium and 0.4 (95% CI 0.2–0.9) for potassium. However, we found an insignificant association between sodium intake and hypertension after we adjusted for covariates, consistent with the SMASH study.

Notably, the significant association of sodium, potassium or their ratio with BP is mainly attributed to patients with hypertension, which is consistent with previous reports and suggests that perhaps salt reduction in hypertensive patients is more effective and worthwhile. In view of the widespread low potassium intake in the Chinese population, potassium supplementation may be prioritized for the general population, including consuming healthier diets of potassium-rich foods such as vegetables and fruits, and low-sodium salt (i.e., potassium-enriched salt substitutes) should be recommended to the general population.

One main finding here is that at higher sodium intake, potassium intake decreased the strength of the sodium-BP relationship^17^. We found that the association of higher sodium excretion with BP was lower among those with higher urinary potassium excretion. Collectively, sodium and potassium, the most abundant extracellular and intracellular cations, respectively, are not easily assessed separately because they are inextricably linked and maintain homeostasis through Na–K-ATPase and interrelationship effects^35^. In the DASH trial, the effects of sodium were modified by the amount of consumed dietary potassium^36^. The linear relationship between increased sodium and potassium intake determines the infeasibility that potassium intake increased while sodium intake decreased dramatically among populations by modifying diets^37,38^. In contrast, similar to the Mediterranean diet, focusing on increasing potassium intake by consuming more potassium-rich foods such as vegetables, fruit and nuts, rather than focusing on decreasing sodium intake, is likely to significantly reduce BP, CVD and mortality^39,40^. Similarly, we found that high sodium excretion combined with low potassium excretion was associated with markedly higher BP than either alone and was associated with substantially higher BP than low sodium excretion combined with high potassium excretion^41^. Moreover, increasing potassium intake might also reduce the occurrence and severity of salt sensitivity^30^. The greater effect of potassium on BP observed in our study may indicate that intervention strategies focusing on increasing dietary potassium consumption in individuals who are potassium deficient or with a high sodium intake could be worthwhile. Improving dietary quality (i.e., via potassium supplementation) is likely easier to achieve than requiring complete control of dietary sodium consumption (low sodium intake < 2000 mg/day), which is impractical in some populations^36,42^.

The mechanisms and pathways involved in the independent and interactive effects of sodium and potassium on BP remain to be confirmed. The strengths of this study include the large representative Chinese population, follow-up completion, the relatively robust method for estimating sodium intake at the population level through one 24-h urine collection with an adequate sample size^43,44^, the rigorous approach to variable measurements, and adjudicated hypertension outcome events. Our findings are subject to the following limitations. First, this study only collected and tested 24-h urine once because of the associated high participant burden and high cost^45^. Given that the large within-individual variability in consumption may be overestimated, the strengths of the associations of sodium and potassium intake with BP may be underestimated^46^. Second, given the observational nature of the study, although we adjusted for major confounders of the association between sodium and potassium excretion and BP, adjustment for these factors may attenuate or amplify the association, and residual confounding is a potential limitation^47–49^. Third, causality in outcomes and exposure cannot be inferred from the cross-sectional survey data in this study. Reverse causation occurs when the likelihood of exposure is causally affected by the research outcome^50^. Persons with hypertension or other pre-existing illnesses (cardiovascular disease, kidney disease) may be advised to reduce sodium intake, and persons treated with diuretics or drugs that block RAAS provoking hyponatremia may have a lower apparent sodium intake^51^.

## Conclusions

These cross-sectional results showed that a significant dose-dependent association between sodium and BP mainly appeared among hypertensive patients, who therefore might benefit from a reduced sodium intake, and a strong inverse association between potassium and BP involved a larger fraction of the population, especially among those with a potassium intake below 1000 mg/24 h, who should probably increase their potassium intake. These results were valuable for policymakers to develop and implement public health strategies of salt reduction and hypertension prevention.

## Supplementary Information

Supplementary Information

## Abbreviations

BP: Blood pressure
SRHPP: Salt reduction and hypertension prevention project
CVD: Cardiovascular disease
CKD: Chronic kidney disease
CDC: Centers for disease control and prevention
SBP: Systolic blood pressure
DBP: Diastolic blood pressure
BMI: Body mass index
SD: Standard deviation
CI: Confidence interval
OR: Odds ratio
WHO: World Health Organization
