# Supplementary material for: Association between 24-h urinary sodium and potassium excretion and blood pressure among Chinese adults aged 18–69 years
Source: Sci Rep. 2021 Feb 10;11:3474. doi: 10.1038/s41598-021-83049-8 (PMC7876040; doi:10.1038/s41598-021-83049-8)
Supplement: Supplementary file 1 — Supplementary Information [file 41598_2021_83049_MOESM1_ESM.doc]

**Association between 24-hour urinary sodium and potassium excretion and blood pressure among Chinese adults aged 18-69 years**

Xiaofu Du^1^, Le Fang^1^, Jianwei Xu^2^, Xiangyu Chen^1^, Yamin Bai^2^, Jieming Zhong^1*^

^1^ Zhejiang Provincial Center for Disease Control and Prevention, 3399 Binsheng Road, Hangzhou 310051, China; xfdu@cdc.zj.cn (X.F.D.); lef@cdc.zj.cn (L.F.); xychen@cdc.zj.cn (X.Y.C.); jmzhong@cdc.zj.cn (J.M.Z.)

^2^ National Center for Chronic and Noncommunicable Disease Control and Prevention, Chinese Center for Disease Control and Prevention, 27 Nanwei Road, Beijing 100050, China; xujianwei@ncncd.chinacdc.cn (J.W.X.); baiyamin@ncncd.chinacdc.cn (Y.M.B.)

**Supplemental Table 1.** Main results from sensitivity analyses among Chinese adults aged 18-69 years, SRHPP 2017.

| Characteristic | Blood Pressure | | |  | Hypertension | | | |
| --- | --- | --- | --- | --- | --- | --- | --- | --- |
|  | SBP | DBP | MBP |  | Q1 (12.5^th^ Percentile) | Q2 (37.5^th^ Percentile) | Q3 (62.5^th^ Percentile) | Q4 (87.5^th^ Percentile) |
|  | β-coefficient per 1000 mg, 95% CI | β-coefficient per 1000 mg, 95% CI | β-coefficient per 1000 mg, 95% CI |  | OR, 95% CI | OR, 95% CI | OR, 95% CI | OR, 95% CI |
| 1) Individuals not taking antihypertensive medications, n=1220 | | | | | | | | |
| Sodium excretion | 0.55 (-0.08 , 1.18) | 0.41* (0.02 , 0.80) | 0.48* (0.01 , 0.95) |  | 1.00 | 1.02 (0.66 , 1.58) | 1.00 (0.64 , 1.56) | 1.25 (0.76 , 2.04) |
| Potassium excretion | -3.54* (-5.14 , -1.94) | -1.32* (-2.31 , -0.33) | -2.43* (-3.63 , -1.22) |  | 1.00 | 0.56* (0.36 , 0.86) | 0.54* (0.34 , 0.85) | 0.51* (0.31 , 0.83) |
| Sodium-to-potassium ratio | 0.78* (0.41 , 1.15) | 0.34* (0.12 , 0.57) | 0.56* (0.29 , 0.84) |  | 1.00 | 1.27 (0.82 , 1.97) | 1.47 (0.96 , 2.28) | 1.95* (1.26 , 3.01) |
| 2) Individuals without CVD, n=1391 | | | | | | | | |
| Sodium excretion | 0.60* (0.00 , 1.20) | 0.41* (0.03 , 0.78) | 0.50* (0.05 , 0.95) |  | 1.00 | 0.96 (0.65 , 1.41) | 0.98 (0.66 , 1.47) | 1.36 (0.87 , 2.12) |
| Potassium excretion | -3.24* (-4.74 , -1.73) | -1.00* (-1.94 , -0.07) | -2.12* (-3.25 , -0.99) |  | 1.00 | 0.54* (0.36 , 0.80) | 0.58* (0.39 , 0.88) | 0.50* (0.32 , 0.78) |
| Sodium-to-potassium ratio | 0.80* (0.44 , 1.15) | 0.31* (0.09 , 0.53) | 0.55* (0.29 , 0.82) |  | 1.00 | 1.43 (0.98 , 2.09) | 1.41 (0.96 , 2.07) | 1.84* (1.24 , 2.72) |
| 3) Excluding alcohol consumption from covariates, n=1424 | | | | | | | | |
| Sodium excretion | 0.65* (0.05 , 1.24) | 0.46* (0.09 , 0.82) | 0.55* (0.11 , 1.00) |  | 1.00 | 0.92 (0.62 , 1.35) | 0.91 (0.61 , 1.35) | 1.26 (0.81 , 1.94) |
| Potassium excretion | -3.08* (-4.57 , -1.58) | -0.96* (-1.88 , -0.04) | -2.02* (-3.13 , -0.90) |  | 1.00 | 0.58* (0.39 , 0.86) | 0.62* (0.41 , 0.92) | 0.54* (0.35 , 0.84) |
| Sodium-to-potassium ratio | 0.78* (0.43 , 1.13) | 0.32* (0.10 , 0.54) | 0.55* (0.29 , 0.81) |  | 1.00 | 1.35 (0.94 , 1.96) | 1.36 (0.93 , 1.98) | 1.69* (1.15 , 2.48) |
| 4) Excluding BMI from covariates, n=1424 | | | | | | | | |
| Sodium excretion | 1.10* (0.48 , 1.71) | 0.77* (0.38 , 1.15) | 0.93* (0.47 , 1.40) |  | 1.00 | 0.94 (0.65 , 1.36) | 1.07 (0.73 , 1.55) | 1.54* (1.02 , 2.34) |
| Potassium excretion | -3.04* (-4.60 , -1.49) | -0.94 (-1.91 , 0.03) | -1.99* (-3.17 , -0.82) |  | 1.00 | 0.64* (0.44 , 0.92) | 0.70 (0.47 , 1.02) | 0.60* (0.39 , 0.91) |
| Sodium-to-potassium ratio | 0.84* (0.47 , 1.21) | 0.36* (0.13 , 0.59) | 0.60* (0.32 , 0.88) |  | 1.00 | 1.49* (1.05 , 2.12) | 1.50* (1.04 , 2.15) | 1.80* (1.24 , 2.61) |

All results presented here are from fully adjusted models, adjusting for age, sex, ethnicity, body mass index, education level, history of cardiovascular disease, mellitus status, chronic kidney disease, antihypertensive medication use, smoking status, alcohol use status and physical activity. In addition, models examining sodium excretion are simultaneously adjusted for potassium excretion, and vice versa. BMI, body mass index; CI indicates confidence interval; and OR, odds ratio.

β-coefficients for sodium and potassium indicate change in mm Hg of blood pressure associated with per 1000 mg difference in excretion; β-coefficient for ratio of sodium-to-potassium indicates change in mm Hg of blood pressure associated with each 1-unit increase in molar ratio.

* Indicates *P*<0.05.
